# Benchmarking the readability, quality, and educational suitability of large language models in communicating pertussis

**DOI:** 10.3389/fpubh.2026.1799204

**Published:** 2026-05-11

**Authors:** Xue Feng Zhong, Rong Liu

**Affiliations:** 1 Department of Clinical Trials of Drugs, Affiliated Jinhua Hospital, Zhejiang University School of Medicine, Jinhua Municipal Central Hospital, Jinhua, China; 2 Department of Pharmacy, The Affiliated Changsha Central Hospital, Hengyang Medical School, University of South China, Changsha, China

**Keywords:** artificial intelligence, large language models, online medical education, pertussis, public health education

## Abstract

**Background:**

Pertussis (whooping cough) is a highly contagious respiratory infection that continues to cause substantial morbidity and mortality, particularly among infants, and has re-emerged globally among adolescents and adults. Large language models (LLMs) are increasingly used for health communication and science popularization; however, evidence regarding their readability, quality, and educational suitability for disease-specific patient education remains limited. To date, no systematic evaluation has focused on LLM-generated pertussis health education materials.

**Objective:**

This study aimed to systematically evaluate and compare the performance of five mainstream LLMs in generating pertussis-related science popularization content, with particular attention to readability, informational quality, and educational suitability.

**Methods:**

A cross-sectional simulation study was conducted using 20 frequently asked pertussis-related questions covering five domains: basic knowledge, symptom presentation, diagnostic methods, treatment and management, and prevention and prognosis. On October 28, 2025, all questions were identically input into five publicly accessible LLMs. Text readability was assessed using seven classical indices. Two independent pharmacists performed blinded evaluations using the Chinese version of the Patient Education Materials Assessment Tool for print materials (C-PEMAT-P) and the Global Quality Score (GQS). Additionally, two independent clinical experts assessed the factual accuracy and guideline concordance of each LLM-generated response against the Chinese Pertussis Diagnosis and Treatment Guidelines (2024) using a 4-point scale. Inter-rater agreement was evaluated using Cohen’s kappa coefficient.

**Results:**

ChatGPT, DeepSeek, and Doubao achieved significantly higher C-PEMAT and GQS scores than Wenxin Yiyan and Gemini (*p* < 0.001), indicating superior understandability, actionability, and overall quality. Median C-PEMAT scores across all models suggested generally acceptable accessibility for patient education. Regarding factual accuracy and guideline concordance, ChatGPT achieved the highest mean score. No harmful advice or direct guideline contradictions were identified in any model output. Correlation analyses showed weak associations between traditional readability metrics and GQS, whereas C-PEMAT demonstrated a moderate positive correlation with GQS (r = 0.34).

**Conclusion:**

Mainstream LLMs show preliminary capability in generating pertussis-related health education materials, although substantial inter-model variability persists. Domain-specific patient education assessment tools better capture perceived informational quality than generic readability metrics. These findings support the cautious, assistive use of LLMs in health communication within a human-AI collaborative framework.

## Introduction

Pertussis, commonly known as whooping cough, is an acute respiratory infectious disease caused by *Bordetella pertussis*. Clinically, it is characterized by paroxysmal coughing episodes often followed by a distinctive inspiratory “whoop.” Transmission occurs primarily via respiratory droplets, and the disease course typically progresses through three stages—catarrhal, paroxysmal, and convalescent—over approximately 2–3 months (1, 2). Infants represent the most vulnerable population, facing elevated risks of severe complications such as pneumonia, encephalopathy, and death (3, 4).

Despite widespread vaccination with pertussis-containing combination vaccines (such as DTaP), a global resurgence of pertussis has been observed in recent years, particularly among adolescents and adults. Waning vaccine-induced immunity, pathogen adaptation, enhanced surveillance, and changes in immunization strategies are considered contributory factors (5). Importantly, pertussis in older age groups often presents atypically, leading to underdiagnosis and facilitating household transmission. Strengthening public awareness, improving early recognition, and enhancing preventive education remain critical components of pertussis control strategies (6, 7).

Health science popularization refers to the translation of specialized medical knowledge into accessible, actionable information for the general public (8). In the context of pertussis, effective popularization can improve vaccination uptake, early symptom recognition, and appropriate healthcare-seeking behavior (9). Large language models (LLMs) have the potential to empower such efforts by providing on-demand, personalized, and simplified explanations of complex medical content, thereby bridging the gap between clinical guidelines and patient understanding. Recent studies have demonstrated that LLMs can generate accurate and comprehensible patient education materials when appropriately prompted, making them promising tools for health communication (1). While certain challenges remain, the overall capabilities of LLMs offer substantial opportunities for improving public health literacy.

Large language models (LLMs), including the Generative Pre-trained Transformer (GPT) series and other contemporary architectures, have rapidly transformed access to medical information. Trained on large-scale general and scientific corpora, LLMs exhibit strong natural language understanding and generation capabilities, enabling conversational interaction, information synthesis, and basic reasoning (1, 10–12). In healthcare contexts, LLM applications broadly fall into two categories: (1) public-facing intelligent health assistants for science popularization and preliminary consultation guidance, and (2) auxiliary tools for healthcare professionals, supporting literature retrieval, documentation drafting, and educational tasks (1, 13).

To date, systematic evaluations of LLM performance in generating disease-specific health science popularization materials remain scarce, particularly for pediatric respiratory infectious diseases such as pertussis. Therefore, we formulated the core hypothesis that mainstream LLMs differ significantly in their ability to generate readable, high-quality, and educationally suitable pertussis health content, and that domain-specific assessment tools better reflect user-perceived quality than generic readability formulas. This study aims to comprehensively assess five mainstream LLMs—ChatGPT, Doubao, DeepSeek, Wenxin Yiyan, and Gemini—in generating pertussis-related Q&A content. Performance is evaluated across three dimensions: readability, information quality, and educational suitability. The findings are intended to inform public health communication, guide clinicians considering AI-assisted patient education, and provide empirical evidence for optimizing LLM deployment in health communication.

## Materials and methods

### Ethical considerations

All data used in this study were generated by LLMs. The research did not involve any human or animal subjects and contained no personally identifiable information or biological samples from individuals. In accordance with prevailing international and relevant Chinese academic ethical guidelines, this type of data-centric study without human participants does not require review or approval by an institutional ethics committee.

### Research procedure

On October 28, 2025, two clinical pharmacists (with 3 and 5 years of clinical work experience) compiled a total of 20 common questions concerning pertussis based on frequently asked questions from patients and caregivers in a tertiary hospital in China. These 20 questions were categorized into five aspects of the disease: basic knowledge, symptom presentation, diagnostic methods, treatment and management, and prevention and prognosis (Table 1). The questions were reviewed by an additional patient educators and a patient for representativeness. Researchers input these 20 questions into five publicly and freely accessible large language models: Doubao, DeepSeek, Wenxin Yiyan, Gemini, and ChatGPT. The exact model versions were: ChatGPT (GPT-5, released August 8, 2025); Gemini (Gemini 2.5 flash, released October 7, 2025); Doubao (Doubao 1.6, version October 2025); DeepSeek (DeepSeek-V3.2-Exp, released September 2025); Wenxin yiyan (Wenxin yiyan 4.5, released April 2025). All prompts were submitted in Simplified Chinese in new, fresh chat sessions with memory cleared and web search disabled. Free tier accounts were used with default safety settings. Each question was submitted once between 9:00–11:00 a.m. on the same day to minimize temporal variability. The exact prompt used for each question, provided in both English and Chinese, was: “Please answer the following question about pertussis in Simplified Chinese at a level suitable for patient education: [question]”.

**Table 1:** List of 20 pertussis-related questions categorized into five domains.

Issue list
1. Disease cognition
1. What is the definition of pertussis, and what are its core pathophysiological mechanisms?
2. What are the main differences between pertussis and common acute upper respiratory tract infections?
3. What are the typical clinical stages of pertussis, and what are the characteristics of each stage?
4. Is pertussis contagious, and what are its main routes of transmission?
2. Symptom performance
1. What is the main pathogen that causes pertussis?
2. What are the key risk factors for the development of pertussis?
3. Why are infants and young children more likely to develop severe pertussis?
4. Does close contact with a pertussis patient significantly increase the risk of infection?
3. Diagnosis and examination
1. What laboratory tests are commonly used to diagnose pertussis?
2. How can pertussis be confirmed based on clinical manifestations and laboratory findings?
3. What is the role of PCR testing in the diagnosis of pertussis?
4. What are the key points in the differential diagnosis between pertussis and other diseases that cause paroxysmal cough?
4. Treatment and management
1. What are the first-line antibiotic treatments for pertussis?
2. How do treatment strategies for pertussis differ among different age groups?
3. What are the clinical treatment strategies for severe or complicated pertussis?
4. What adverse reactions may occur with commonly used medications for pertussis?
5. Prevention and prognosis
1. What are the key public health measures for preventing the transmission of pertussis?
2. What are the vaccination strategies and protective effects of pertussis vaccines (such as DTaP / Tdap)?
3. What care and lifestyle management should be considered during the recovery period of children with pertussis?
4. How should treatment effectiveness be evaluated after pertussis therapy, and how can recurrence or further transmission be prevented?

The questions were developed based on frequently asked questions from patients and caregivers in a tertiary hospital in China, then reviewed by a patient educator and a pertussis patient for representativeness. The five domains correspond to the content categories used in Table 4.

### Accuracy analysis

To assess factual accuracy, two independent experts (a senior attending physician in the Department of Internal Medicine with over 10 years of clinical experience, and a senior physician in the Department of Infectious Diseases with 6 years of clinical experience) rated each LLM response against the Chinese Pertussis Diagnosis and Treatment Guidelines (2024) and CDC pertussis guidelines (2025) (14, 15). A 4-point concordance scale was used: 0 = contradicts guideline, 1 = omits key points, 2 = generally consistent, 3 = fully consistent. Inter-rater agreement was excellent (*κ* = 0.82).

### Readability evaluation

We employed various calculation formulas provided by an online text readability assessment tool^1^ to analyze the answers generated by the LLMs (Table 2). As there is currently no authoritative data indicating which readability metric is more accurate or reliable, nor an established gold standard, this study adopted the mainstream set of indicators widely used in previous literature (16, 17).

**Table 2:** Readability tools, formulas and descriptions.

Readability index	Description	Formula
Gunning FOG (GFOG)	It estimates the number of years of education required for a person to understand a given text.	G = 0.4 X (W/S + ((C*W) X 100))
Flesch Reading Ease Score (FRES)	It was created to assess the readability of newspapers and is particularly effective for evaluating school textbooks and technical manuals. The scores range from 0 to 100, with higher scores indicating greater ease of reading.	I = (206.835 – (84.6 X (B/W)) – (1.015 X (W/S)))
Flesch–Kincaid grade level (FKGL)	Delineates the academic capacity level imperative for grasping the written material	G = (11.8 X (B/W)) + (0.39 X (W/S)) – 15.59
Simple Measure of Gobbledygook (SMOG)	It measures the number of years of education the average person needs to understand a text.	G = 1.0430 X √C + 3.1291
Coleman–Liau (CL) score	Evaluates the educational level required for understanding a text and offers an associated grade level in the US education system.	G = (−27.4004 X (E/100)) + 23.06395
Linsear Write (LW)	Offers an approximate assessment of the academic level needed to comprehend the text.	LW = (R + 3C)/S Result • If >20, divide by 2 •If ≤20, subtract 2, and then divide by 2
Automated readability index (ARI)	Assesses the scholastic rank in American educational institutions needed to be capable of comprehending written material. The greater the number of characters, the more complex the term.	ARI = 4.71 X I + 0.5*ASL – 21.43

Seven classical readability indices were used to assess the LLM-generated texts. G = Grade level; B=Number of syllables; W=Number of words; S=Number of sentences; I=Flesch Index Score; SMOG = Simple Measure of Gobbledygook; C=Complex words (≥3 syllables); E = predicted Cloze percentage = 141.8401 – (0.214590 X number of characters) + (1.079812*S); C* = Complex words with exceptions including, proper nouns, words made 3 syllables by addition of “ed” or “es,” compound words made of simpler words. ASL = the average number of sentences per 100 words R = the number of words ≤2 syllables.

### Quality assessment

This study utilized the C-PEMAT-P scale (Chinese version of the Patient Education Materials Assessment Tool) and the GQS (Global Quality Score) scale to determine the reliability of the textual answers (18–21). The C-PEMAT-P contains 24 items, divided into two dimensions: “Understandability” (16 items, including logical organization of information, simplification of professional terminology, etc.) and “Actionability” (8 items, including provision of concrete action guidance, suitability for target populations, etc.). Each item is scored on a 0–1 scale (0 = completely does not meet, 1 = completely meets), with a total score ranging from 0 to 24. A higher score indicates greater accessibility of the material for users. The GQS uses a 1–5 point scale: 1 corresponds to “Poor quality, logically confused content, lacking key information, and of no practical value to users”; 2 corresponds to “Generally poor quality, content with insufficient logic, incomplete core information, and limited practical value”; 3 corresponds to “Moderate quality, important information partially discussed and possessing basic practical value”; 4 corresponds to “Good quality, logically clear content covering most relevant information and of high practical value”; 5 corresponds to “Excellent quality, logically rigorous and well-coherent content with significant practical value for users.” On October 28, 2025, two independent pharmacists assessed the materials using the aforementioned scales. The evaluators were blinded to model identity: all 100 responses (20 questions × 5 models) were randomized and anonymized with random codes. When scoring discrepancies arose, a third expert (senior attending physician, 12 years of experience) adjudicated the final rating, and reviewers reached consensus through in-depth discussion, the final score recorded was the consensus score. This study used Cohen’s Kappa coefficient to quantify inter-rater agreement, with the following interpretation standards: *κ* > 0.75 indicates excellent agreement, 0.40 ≤ κ ≤ 0.75 indicates acceptable agreement, and κ < 0.40 reflects poor agreement. All disagreements were resolved through consensus discussion to ensure the rigor and reliability of the assessment. It was verified that the Cohen’s Kappa coefficients for both the C-PEMAT-P and GQS scales were greater than 0.75, meeting the standard for excellent agreement.

### Statistical analysis

We used the Shapiro–Wilk test to assess normality for each variable. For measurement variables with a normal distribution (such as the C-PEMAT-P score and GQS score, presented as mean ± standard deviation [Mean ± SD]), one-way analysis of variance (ANOVA) was used for comparisons among multiple groups38. For variables with a non-normal distribution (such as ARI and FRES, presented as median and interquartile range [M, Q1, Q3]), the Kruskal–Wallis H test was applied to assess differences among groups. A two-sided *p* value < 0.05 was considered statistically significant. All data analyses and data visualizations were conducted using Python 3.14. The correlation matrix was computed using the Pandas library, and the heatmap was generated using the Seaborn library, where each cell represents the pairwise *r* value. Color intensity indicates the strength and direction of correlation (red: positive, blue: negative).

## Results

### Normality analysis

C-PEMAT and GQS scores approximated normality (Shapiro–Wilk *p* > 0.05), whereas all readability indices (ARI, FRES, GFOG, FKGL, CL, SMOG, LW) showed non-normal distributions (*p* < 0.05), showed that C-PEMAT and GQS scores were approximately normally distributed (*p* > 0.05), while all readability indices exhibited non-normal distributions (*p* < 0.05).

### Accuracy analysis

Factual accuracy against guidelines (Table 3): The mean concordance scores (0–3) were: ChatGPT 2.85 ± 0.41, DeepSeek 2.63 ± 0.57, Doubao 2.42 ± 0.60, Wenxin Yiyan 1.77 ± 0.74, Gemini 1.56 ± 0.61. No response contained harmful advice or direct contradictions to guidelines. However, Wenxin yiyan and Gemini had more omissions of key information.

**Table 3:** Performance comparison of five large language models across concordance scores, C-PEMAT, GQS, and readability indices.

Variables	Total (*n* = 100)	Doubao (*n* = 20)	Deep seek (*n* = 20)	Wenxin Yiyan (*n* = 20)	Gemini (*n* = 20)	GPT-5 (*n* = 20)	Statistic	*p*
Concordance scores, Mean ± SD	1.71 ± 0.95	2.42 ± 0.60	2.63 ± 0.57	1.77 ± 0.74	1.56 ± 0.61	2.85 ± 0.41	*F* = 9.27	**0.007**
C-PEMAT score, Mean ± SD	7.99 ± 2.17	8.35 ± 1.73	8.45 ± 1.88	7.25 ± 2.07	6.75 ± 2.57	9.15 ± 1.79	*F* = 4.58	**0.002**
GQS score, Mean ± SD	3.08 ± 1.24	3.50 ± 0.76	3.75 ± 0.55	1.80 ± 0.52	1.80 ± 0.41	4.55 ± 0.60	*F* = 89.72	**<0.001**
ARI, M (Q1, Q3)	16.90 (15.20, 19.47)	18.32 (15.16, 20.46)	15.91 (15.41, 25.69)	15.21 (13.84, 16.47)	19.49 (16.93, 21.16)	16.84 (15.17, 18.16)	χ^2^ = 20.06#	**<0.001**
FRES, M (Q1, Q3)	22.00 (11.00, 35.00)	27.00 (17.00, 35.75)	18.00 (0.00, 34.25)	24.50 (18.50, 40.25)	12.00 (4.50, 26.00)	28.50 (17.00, 34.00)	χ^2^ = 10.70#	**0.03**
GFOG, M (Q1, Q3)	15.80 (13.62, 17.68)	15.05 (12.15, 16.60)	16.05 (14.40, 20.10)	15.45 (12.97, 17.20)	17.15 (15.88, 18.82)	14.30 (12.70, 17.32)	χ^2^ = 13.98#	**0.007**
FKGL, M (Q1, Q3)	15.77 (13.42, 17.74)	16.08 (13.77, 17.86)	15.25 (13.00, 22.38)	14.59 (12.62, 16.15)	17.84 (15.91, 19.00)	15.07 (12.99, 16.30)	χ^2^ = 13.54#	**0.009**
CL, M (Q1, Q3)	16.29 (14.87, 18.05)	15.32 (13.84, 16.71)	17.16 (15.47, 18.85)	15.27 (14.53, 16.39)	17.34 (16.19, 18.49)	16.81 (15.30, 17.93)	χ^2^ = 11.22#	**0.024**
SMOG, M (Q1, Q3)	13.60 (11.98, 15.23)	13.96 (11.57, 15.29)	13.07 (11.98, 19.25)	12.46 (11.15, 14.11)	15.23 (13.63, 16.54)	12.91 (11.27, 14.28)	χ^2^ = 15.80#	**0.003**
LW, M (Q1, Q3)	55.00 (52.00, 61.00)	55.00 (53.00, 60.50)	56.50 (48.50, 61.25)	57.50 (53.75, 63.25)	53.00 (50.00, 54.75)	59.50 (54.00, 65.00)	χ^2^ = 10.27#	**0.036**

Values are presented as mean ± SD for normally distributed variables (concordance scores, C-PEMAT, GQS) or median (Q1, Q3) for non-normally distributed readability indices. ANOVA was used for normally distributed variables, and Kruskal-Wallis H test for non-normally distributed variables. Bolded *p*-values indicate statistically significant differences (*p* < 0.05). F: ANOVA, #: Kruskal-waills test SD: standard deviation, M: Median, Q1: 1st Quartile, Q3: 3st Quartile.

### Readability analysis

This study systematically examined differences in text quality and readability of pertussis-related health education materials generated by different large language models across two dimensions: model type and content topic. First, five mainstream LLMs (Doubao, Deep Seek, Wenxin Yiyan, Gemini, and ChatGPT) were compared with respect to patient education suitability, as assessed by the C-PEMAT, overall quality evaluated using the Global Quality Score (GQS), and multiple readability indices. Second, variations in these metrics were analyzed across five categories of health education topics: Disease Cognition, Symptom Performance, Diagnosis and Examination, Treatment and Management, and Prevention and Prognosis.

Inter-model comparisons (Table 3) demonstrated significant differences in both C-PEMAT and GQS scores. ChatGPT, DeepSeek, and Doubao achieved significantly higher C-PEMAT scores (9.15 ± 1.79, 8.45 ± 1.88, and 8.35 ± 1.73, respectively) and GQS scores (4.55 ± 0.60, 3.75 ± 0.55, and 3.50 ± 0.76, respectively) than Wenxin Yiyan and Gemini (all *p* < 0.001). These findings indicate that content generated by these three models exhibited greater understandability, actionability, and overall presentation quality. In addition, significant inter-model differences were observed across multiple readability indices, including ARI, FRES, GFOG, FKGL, SMOG, lexical word ratio (LW), and the Coleman–Liau Index (CL).

Analysis by content topic (Table 4) revealed significant differences in C-PEMAT scores across the five topic categories, whereas no statistically significant differences were observed in GQS scores. With respect to readability metrics, materials addressing Diagnostic Methods, Treatment and Management, and Prevention and Prognosis consistently demonstrated higher scores on several reading difficulty indices (such as ARI and FKGL) compared with texts focusing on Basic Disease Knowledge and Symptom Presentation, indicating greater linguistic complexity. The Flesch Reading Ease Score further indicated that Basic Disease Knowledge content exhibited the highest readability, whereas Treatment and Management content was the most difficult to read. Similar patterns were observed for the Gunning Fog Index, SMOG index, and Coleman–Liau Index.

**Table 4:** Analysis results by content category across five pertussis education domains.

Variables	Total (*n* = 100)	Disease cognition dimension (*n* = 20)	Symptom performance dimension (*n* = 20)	Diagnosis and examination dimension (*n* = 20)	Treatment and management dimension (*n* = 20)	Prevention and prognosis dimension (*n* = 20)	Statistic	*p*
C-PEMAT score, Mean ± SD	7.99 ± 2.17	6.40 ± 1.57	7.15 ± 1.46	7.50 ± 2.37	8.85 ± 2.16	10.05 ± 0.89	*F* = 13.49	**<0.001**
GQS score, Mean ± SD	3.08 ± 1.24	3.00 ± 1.34	3.20 ± 1.32	3.05 ± 1.23	2.95 ± 1.28	3.20 ± 1.15	*F* = 0.17	0.955
ARI, M (Q1, Q3)	16.90 (15.20, 19.47)	15.83 (14.86, 16.95)	15.81 (14.77, 17.52)	18.16 (15.58, 19.48)	17.87 (16.69, 20.02)	18.51 (15.24, 21.24)	χ^2^ = 9.49#	0.05
FRES, M (Q1, Q3)	22.00 (11.00, 35.00)	33.50 (23.75, 38.25)	26.00 (10.75, 40.25)	23.50 (17.00, 30.25)	11.50 (8.00, 16.75)	20.00 (10.25, 32.75)	χ^2^ = 16.40#	**0.003**
GFOG, M (Q1, Q3)	15.80 (13.62, 17.68)	14.25 (12.38, 15.12)	14.30 (12.17, 17.90)	15.80 (14.50, 17.20)	17.55 (17.10, 18.85)	16.50 (14.70, 18.23)	χ^2^ = 20.03#	**<0.001**
FKGL, M (Q1, Q3)	15.77 (13.42, 17.74)	14.41 (12.14, 15.05)	14.97 (12.67, 16.77)	16.12 (14.98, 17.95)	16.75 (15.89, 17.98)	15.60 (13.78, 18.63)	χ^2^ = 12.91#	**0.012**
CL, M (Q1, Q3)	16.29 (14.87, 18.05)	15.16 (14.40, 16.61)	16.00 (14.66, 17.85)	15.30 (13.74, 16.34)	18.01 (16.81, 19.12)	17.20 (15.53, 19.81)	χ^2^ = 24.63#	**<0.001**
SMOG, M (Q1, Q3)	13.60 (11.98, 15.23)	12.26 (11.13, 13.13)	12.96 (11.27, 15.32)	13.96 (13.21, 15.95)	14.36 (13.70, 15.41)	13.86 (12.22, 16.54)	χ^2^ = 16.46#	**0.002**
LW, M (Q1, Q3)	55.00 (52.00, 61.00)	58.50 (53.75, 63.25)	54.00 (48.50, 60.25)	56.50 (53.00, 59.75)	55.00 (53.00, 61.00)	53.50 (49.75, 61.00)	χ^2^ = 4.20#	0.379

The five domains (Disease Cognition, Symptom Performance, Diagnosis and Examination, Treatment and Management, Prevention and Prognosis) correspond to the question categories in Table 1. C-PEMAT and GQS scores are shown as mean ± SD; readability indices are shown as median (Q1, Q3). ANOVA was used for C-PEMAT and GQS; Kruskal-Wallis H test for readability indices. Statistically significant differences are indicated in bold (*p* < 0.05). F: ANOVA, #: Kruskal-waills test, SD: standard deviation, M: Median, Q1: 1st Quartile, Q3: 3st Quartile.

### Quality analysis

**Figure 1:**
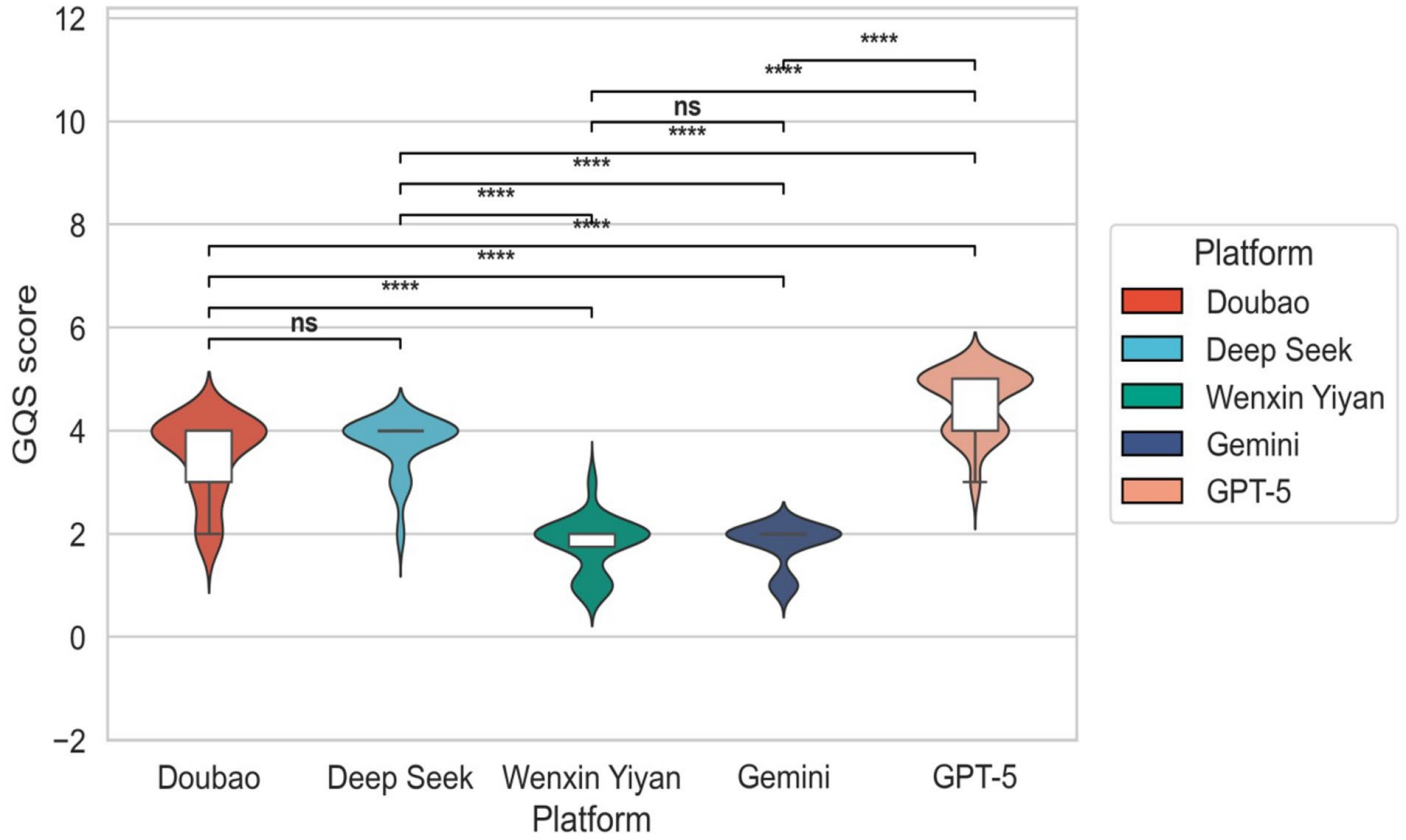
GQS scores of different large models.

Significant differences in GQS scores were observed among the evaluated models (Figure 1). ChatGPT exhibited the highest median GQS score with the narrowest distribution, indicating a strong ability to consistently generate high-quality health information. DeepSeek demonstrated a slightly lower but still relatively high median score with a compact distribution, suggesting competitive performance. Wenxin Yiyan and Gemini showed similar median GQS scores; however, Wenxin Yiyan displayed a wider score distribution with the presence of low-score outliers, indicating variability in output quality. Doubao showed the lowest relative median GQS score and a more dispersed distribution compared to ChatGPT and DeepSeek.

**Figure 2:**
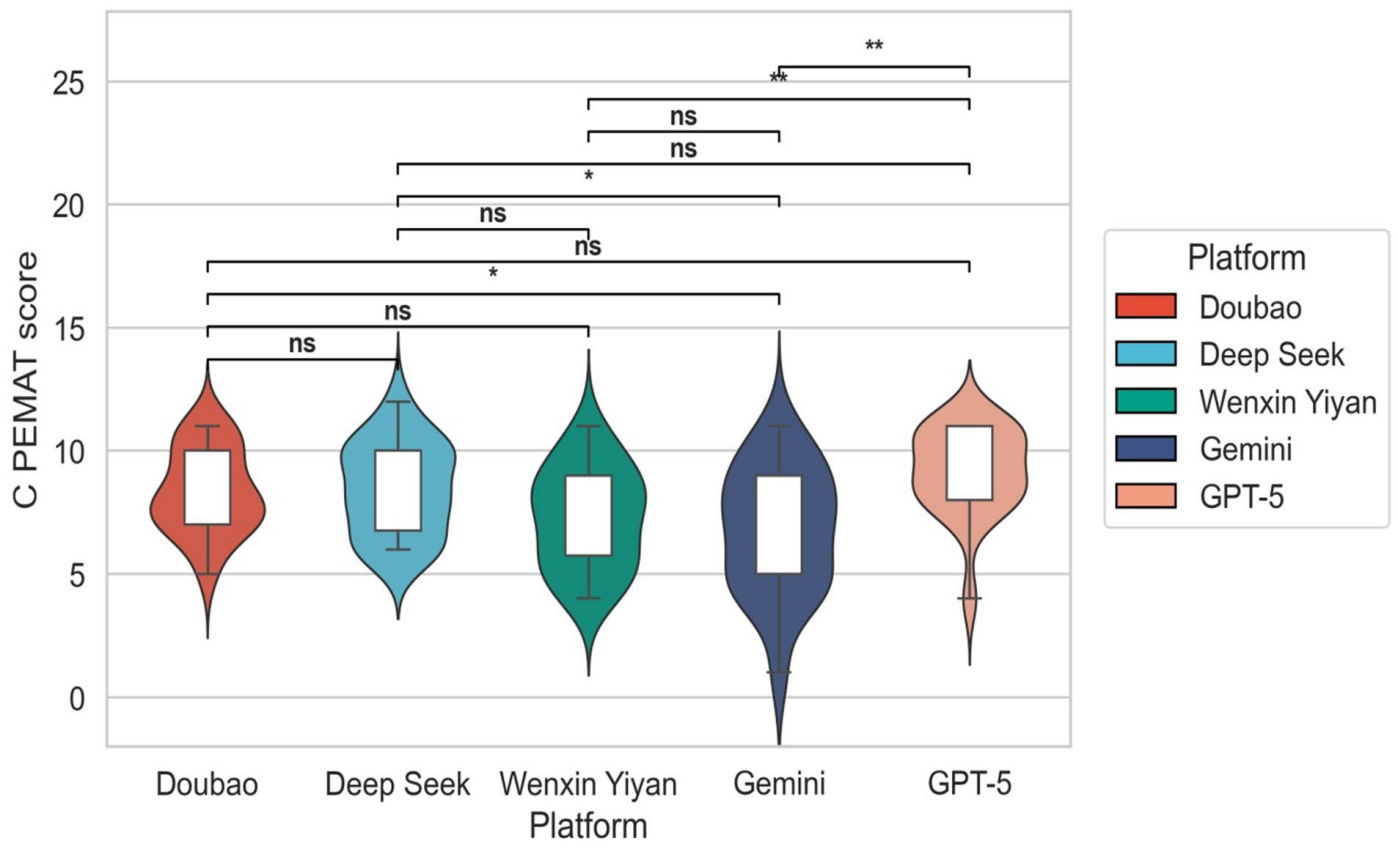
C-PEMAT-P scores of different large models.

With respect to C-PEMAT scores (Figure 2), the median values for all models fell within the range of 8–10 points, suggesting that mainstream LLMs generally possess a baseline capability to generate structurally clear and logically coherent patient education materials. Among the models, ChatGPT achieved the highest median score with the most concentrated distribution, indicating the most robust overall performance. DeepSeek and Wenxin Yiyan followed, whereas Doubao demonstrated the lowest median C-PEMAT score among ChatGPT, DeepSeek, and Doubao, yet still outperformed Wenxin yiyan and Gemini. Its bimodal distribution reflected instability in understandability and actionability.

### Correlation analysis

**Figure 3:**
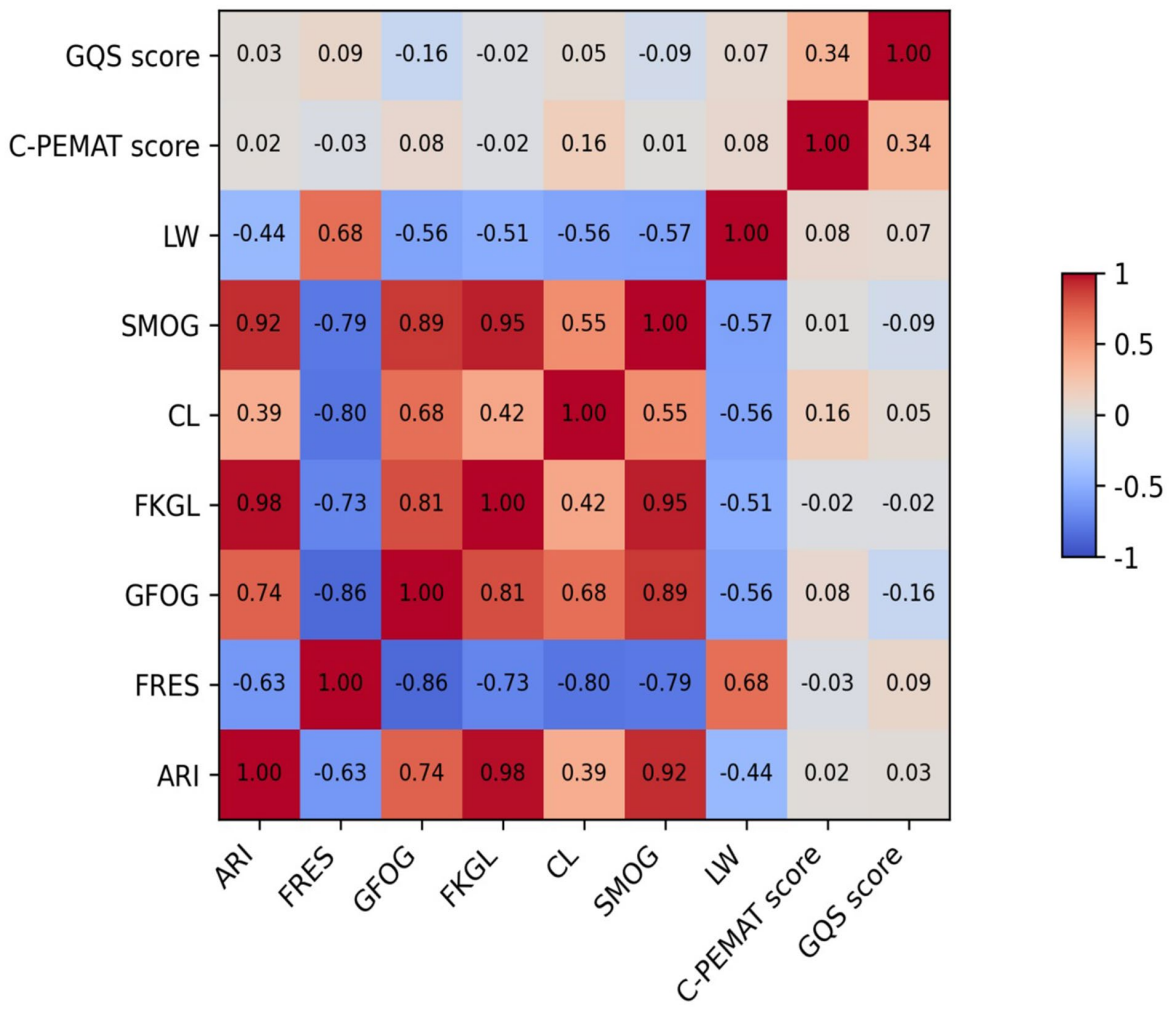
Heatmap of correlations among different indicators.

Correlation analyses were conducted to explore relationships among readability indices, C-PEMAT scores, and user-perceived quality as measured by GQS (Figure 3). Readability ease indicators, such as the Flesch Reading Ease Score, showed a weak positive correlation with GQS (r = 0.09) and strong negative correlations with several reading difficulty indices (GFOG, r = −0.86; SMOG, r = −0.79). Traditional reading difficulty formulas (ARI, FKGL, SMOG, and related indices) were highly intercorrelated (r > 0.89) but demonstrated weak or negligible correlations with GQS (such as ARI vs. GQS, r = 0.03). In contrast, the C-PEMAT score exhibited a moderate positive correlation with GQS (r = 0.34). Lexical richness, as measured by LW, was positively correlated with FRES (r = 0.68) and negatively correlated with multiple reading difficulty indices.

## Discussion

This study presents a systematic, multi-dimensional evaluation of mainstream large language models (LLMs) in the context of pertussis-related health science communication. By jointly examining model type and content category, our findings elucidate key determinants of text quality and readability, thereby providing empirical evidence to support the standardized and responsible integration of LLMs into patient education and public health communication.

### Determinant role of model type in text quality and mechanisms analysis

Our results demonstrate pronounced inter-model variability in the quality of AI-generated pertussis health education materials, with model type emerging as the dominant determinant. Across core quality metrics—suitability for patient education (C-PEMAT) and overall informational quality (GQS)—GPT-5 achieved the highest scores, followed by DeepSeek and Doubao, whereas Wenxin Yiyan and Gemini consistently underperformed. These differences were statistically robust (all *p* < 0.001), underscoring that architectural design, training data composition, and alignment strategies substantially influence the capacity of LLMs to generate clinically appropriate and patient-centered educational content (22).

Beyond mean performance, output stability represents a critical dimension for real-world applicability. High-performing models, particularly GPT-5 and DeepSeek, exhibited narrower interquartile ranges and fewer low-score outliers in both GQS and C-PEMAT distributions, indicating a more reliable ability to meet baseline standards of medical communication across heterogeneous question types (23–25). In contrast, wider score dispersion and frequent low-quality outputs observed in other models highlight concerns regarding robustness and scenario adaptability—factors that pose significant barriers to safe deployment in clinical and public-facing educational settings (26, 27).

### Modulating effect of content category on text readability and its clinical implications

In addition to model effects, our findings reveal a clear topic-dependent pattern in text readability. Across multiple readability indices (ARI, FRES, SMOG, among others), linguistic complexity increased systematically with the intrinsic specialization of content domains. Texts addressing Treatment and Management, as well as Diagnosis and Examination, consistently demonstrated higher reading difficulty, reflecting the necessity to convey complex therapeutic strategies, pharmacological mechanisms, and procedural information (28, 29). In contrast, materials focused on Basic Disease Knowledge and Symptom Presentation exhibited greater readability (30).

These findings carry important implications for clinical communication and public health education. They confirm that content category functions as an independent regulator of information accessibility. Accordingly, when deploying LLMs for health science popularization, the inherent linguistic demands of different topics must be explicitly anticipated. For treatment-related content, deliberate simplification strategies—such as segmenting information into shorter units, defining technical terms, and employing analogies—are essential to preserve comprehension. Conversely, preventive and daily care guidance may prioritize conciseness and directness without compromising clarity (31).

Notably, while content category exerted a strong influence on readability metrics, it did not significantly affect core quality indicators (C-PEMAT and GQS). This suggests that high-performing models are capable of maintaining accuracy, logical coherence, and actionability across topics of varying complexity (32). From a practical standpoint, this finding supports the flexible use of LLMs as assistive tools across diverse patient education scenarios, including initial disease education, interpretation of diagnostic findings, and explanation of treatment plans, provided that appropriate oversight is maintained (33).

### Features and optimization paths for text quality and readability

Correlation analyses further clarify the nuanced relationship between textual readability and perceived quality. Traditional readability formulas exhibited weak direct associations with both C-PEMAT and GQS, reinforcing the notion that superficial linguistic simplicity alone does not equate to effective health communication. Texts composed of short sentences and simple words may still fail if they lack logical structure, essential content, or actionable guidance (34).

Nevertheless, several correlation patterns offer valuable insights for optimization. First, the positive association between GQS and lexical length suggests that a minimum level of informational completeness is necessary to convey rigorous and trustworthy health information. Excessive brevity risks omitting critical elements such as contraindications or stepwise recommendations. Second, the observed balance between lexical complexity and educational suitability highlights a central tension in medical communication: professional terminology is often indispensable for scientific accuracy, yet uncontextualized jargon undermines patient comprehension. Effective health education, therefore, depends on the judicious introduction and clear explanation of technical terms rather than their wholesale elimination (12, 13, 34).

Based on these findings, future optimization of LLMs for health science communication should prioritize: (1) dynamic, personalized content generation that adapts readability to user characteristics such as health literacy and age; (2) multi-objective training frameworks that jointly optimize accuracy, coherence, actionability, and linguistic accessibility; and (3) clearly defined human–AI collaboration boundaries, with explicit prompts for professional consultation in complex or high-risk scenarios. In this framework, LLMs should be positioned as supportive tools that augment, rather than replace, clinician-led health education.

### Practical implications for pertussis health science popularization

Our findings have direct implications for pertussis education. Clinicians and public health educators can consider using ChatGPT, DeepSeek, or Doubao to generate initial drafts of educational materials, particularly for basic knowledge and symptom domains where these models perform well. However, for treatment and diagnosis content, human review is essential to ensure accuracy and appropriate simplification. The moderate correlation between C-PEMAT and perceived quality suggests that patient education materials should be evaluated using domain-specific tools rather than relying solely on readability formulas. This can help improve pertussis awareness among populations with low health literacy.

## Limitations

This study systematically evaluated the quality and readability of LLM-generated pertussis health education texts, but several limitations should be acknowledged. First, the sample was constrained to 20 questions across five topic domains and five LLMs, limiting generalizability to other models and real-world patient queries. Additionally, beyond sample constraints, the evaluation framework relied primarily on expert-assessed objective indicators rather than direct measurement of patient-centered outcomes—such as comprehension, information retention, or behavioral implementation—nor did it incorporate real-time feedback from clinical practitioners. Furthermore, building on these methodological considerations, patient-level heterogeneity—including variations in health literacy, age, and cultural background—was not addressed, thereby precluding the formulation of personalized adaptation strategies and restricting the applicability of findings across diverse populations. There is currently a lack of widely accepted and validated readability assessment instruments specifically designed for Chinese medical patient education materials. Although tools such as the Chinese Readability Index Explorer (CRIE) exist, they have been primarily validated on general educational corpora rather than on medical or health communication content.

The single-run, single-day snapshot design does not account for intra-model variability or platform updates over time. Although a sensitivity analysis showed low variability, future studies should incorporate multiple repetitions. The use of a convenience sample (20 questions) without formal power calculation is another limitation. Finally, the factual accuracy assessment, while newly added, was based on Chinese and US guidelines; generalizability to other countries’ guidelines may be limited.

The medical question-and-answer domain imposes exceptionally high standards for accuracy, safety, and reliability. LLMs face persistent challenges, including hallucinated content, embedded training biases, limitations in complex clinical reasoning, and unresolved issues of accountability and regulation (10). Addressing these challenges requires fine-tuning with high-quality medical data, integration with authoritative evidence-based knowledge sources, robust output verification mechanisms, and human-in-the-loop system designs (35, 36).

### Future directions

Based on the findings of this study, three priority directions for future research are proposed. First, subsequent investigations should expand the scope of evaluated models to include emerging LLMs and medical domain-specific variants fine-tuned on clinical corpora, alongside systematic optimization of prompt engineering strategies and few-shot learning techniques to enhance output quality for complex medical topics.

Second, prospective validation in real-world clinical and community settings is essential to assess how LLM-generated educational materials influence patient comprehension, health behaviors, and clinical outcomes. Such studies should incorporate multi-turn dialogue scenarios and longitudinal tracking to evaluate performance trajectories over time, thereby providing evidence for sustained effectiveness.

Third, future work must prioritize the development of robust implementation frameworks that integrate automated fact-checking mechanisms against evidence-based clinical guidelines, culturally adapted assessment tools, and clearly defined human-AI collaboration boundaries. These frameworks should address not only technical performance but also accountability, transparency, and equity in access to AI-enhanced health education, ensuring these technologies serve as safe and effective adjuncts to professional medical guidance.

## Conclusion

In summary, this study demonstrates that model selection is crucial in ensuring the quality and Suitability of AI-generated health science content, while the topic of the content plays a significant role in shaping its readability. By elucidating the interplay between model characteristics, topic complexity, and communication effectiveness, our findings offer actionable guidance for the responsible deployment of LLMs in patient education and public health communication. Future development should emphasize both informational rigor and adaptive simplification, with the ultimate goal of delivering accurate, accessible, and personalized AI-assisted health education that is tailored to individual needs.

## Data Availability

The original contributions presented in the study are included in the article/supplementary material, further inquiries can be directed to the corresponding author.

## Footnotes

1
https://readabilityformulas.com/
